# Clinical importance in Alzheimer’s disease: effects of anchor agreement and disease severity

**DOI:** 10.1007/s40520-023-02643-0

**Published:** 2024-01-24

**Authors:** Marta Stojanovic, Cynthia Mikula, Samantha John, Andrew Kiselica

**Affiliations:** 1 Department of Psychological and Brain Sciences, Washington University in St. Louis, One Brookings Drive, Box 1125, St. Louis, MO 63130 USA; 2 Institute of Human Nutrition, Columbia University, New York, NY 10032 USA; 3 Department of Brain Health, University of Nevada-Las Vegas, Las Vegas, Nevada, 89154 USA; 4 Department of Health Psychology, University of Missouri-Columbia, Columbia, MO 65211 USA

**Keywords:** Dementia, Alzheimer’s disease, Clinical trials, Minimal clinically important difference

## Abstract

**Objectives:**

Methods of evaluating clinically meaningful decline are critical in research on Alzheimer’s disease. A common method of quantifying clinically meaningful change is to calculate an anchor-based minimal clinically important difference (MCID) score. In this approach, individuals who report a meaningful change serve as the “anchors”, and the mean level of change for this group serves as the MCID. In research on Alzheimer’s disease, there are several possible anchors, including patients, knowledgeable observers (e.g., a family member), and clinicians. The goal of this study was to examine the extent to which agreement among anchors impacts MCID estimation and whether this relationship is moderated by cognitive severity status.

**Methods:**

Analyses were completed on a longitudinal sample of 2247 adults, aged 50–103, from the Uniform Data Set. Outcome measures included the Montreal Cognitive Assessment, Clinical Dementia Rating—Sum of Boxes, and Functional Activities Questionnaire.

**Results:**

For all of the outcomes, the MCID estimate was significantly higher when meaningful decline was endorsed by all of the anchors compared to when there was disagreement among the anchors. In addition, the MCID estimate was higher with increasing severity of cognitive impairment. Finally, cognitive severity status moderated the influence of agreement among anchors on MCID estimation; as disease severity increased, anchor agreement demonstrated less influence on the MCID.

**Conclusions:**

MCID estimates based on one anchor may underestimate meaningful change, and researchers should consider the viewpoints of multiple anchors in constructing MCIDs, particularly in the early stages of cognitive decline.

**Supplementary Information:**

The online version contains supplementary material available at 10.1007/s40520-023-02643-0.

## Introduction

In Alzheimer's disease (AD) research, changes in symptoms are often reported in terms of statistically significant differences on outcome measures, such as Alzheimer’s Disease Assessment Scale cognitive subscale, Clinician’s Interview Based Impression of Change, and Mini Mental Status Exam [1–5]. However, statistically significant differences are not always clinically relevant. Clinical significance indicates whether a decline or improvement in a test score corresponds with a meaningful change from a patient’s perspective [6]. Measures that go beyond statistical significance and quantify decline or improvement in symptoms of the disease are needed to characterize disease progression. This goal is particularly important, given recent debates as to the clinical promise of newly approved pharmacological treatments for Alzheimer’s disease [7].

Minimal clinically important difference (MCID) statistics are a way of quantifying changes in disease symptoms that do not rely on statistical significance alone [8]. An MCID is defined as the smallest change on an outcome measure that corresponds to an added benefit in a patient’s life [9]. Andrews et al. [9] used data from the National Alzheimer’s Coordinating Center Uniform Data Set (UDS) to provide MCID estimates for the Mini Mental Status Exam (MMSE) [10], the Clinical Dementia Rating Sum of Box Scores (CDR-SB) [11], and the Functional Activities Questionnaire (FAQ) [12], which are measures of overall cognitive and functional abilities commonly applied in AD research. Across the outcomes, they found that, as the stage of AD got more severe, the MCID estimate became larger. Overall, a 1–3 point decrease in the MMSE, a 1–2 point increase on the CDR-SB, and a 3–5 point increase on the FAQ corresponded with a meaningful clinical decline, depending on disease severity [9]. Another study, conducted in the ADC-008 data sample with individuals with mild cognitive impairment, found a 1–2.5 point increase in CDR-SB corresponded with a meaningful clinical decline based on a clinician’s response [13].

Since the publication of the Andrews et al. [9] article, important updates have been made to the UDS, on which the analyses were performed. Thousands of additional participants have been added to this database, and there have been some important changes to the primary measures given to participants [14]. For instance, the MMSE has been replaced by the Montreal Cognitive Assessment (MoCA) [15]. Thus, there is a need to extend the work of Andrews et al. [9] and provide MCID estimates for the newly added scale.

### Anchor choice and MCID

The most common way of computing MCID is to use an anchor-based approach [16]. In this method, an anchor (usually participant/patient, clinician, or study partner), provides a subjective judgment about whether there has been a meaningful improvement or decline in symptoms. This subjective judgment is used as the “anchor” by which the MCID is estimated. Typically, this estimation is accomplished by calculating the mean level of change in a measure score for those with an anchor indicating meaningful change. The MCID is calculated using a single anchor, and anchor choice varies widely across MCID studies in the dementia literature [17]. In the Andrews et al. [9] study, the researchers only calculated MCID using clinicians to provide the anchor response. However, in the UDS, participants, study partners, and clinicians are all asked to indicate whether a subject has had a decline in memory relative to previous abilities [14]. Directions for clinicians indicate that judgments should be based on information obtained through the participant, co-participant, medical records, and observations, not neuropsychological testing or neuroimaging (with the exception that the result of the MoCA can be used to aid this judgement) [14].

An alternative to using a single anchor for the MCID might be to use information from these multiple anchors. In a prior publication, we found that, in cognitively normal participants, agreement across clinicians, participants, and study partners was at best moderate (kappa range = 0.24–0.53) when reporting on subjective cognitive problems and neurobehavioral symptoms [18]. However, in cases where agreement occurs, predictive accuracy tends to increase. For example, combining self-reported and study partner-reported information about cognition was found to increase predictive power in explaining conversion to dementia and neuropathological outcomes among individuals with mild cognitive impairment [19–22]. Furthermore, anchor choice may interact with disease severity to influence MCID estimation. Prior studies have found that patient-reported symptoms became less predictive of objective cognitive impairment as disease progressed, whereas study partner-reported symptoms became more predictive [23, 24]. This finding was attributed to a loss of insight about symptoms (i.e., anosognosia) among individuals with AD. Thus, further research is needed to understand the influence of anchor agreement on MCID estimation in the context of AD severity.

### Current study

The aims of this study were to (1) provide updated MCID estimates for measures of cognitive and functional outcomes (MoCA, CDR-SB, and FAQ) using the latest version of the Uniform Data Set; (2) assess the influence of anchor agreement on MCID estimation; and (3) examine whether the effect of anchor agreement on MCID estimation is moderated by disease severity. It was hypothesized that there would be a main effect of agreement on MCID estimation, such that the MCID would increase when there was agreement. Similarly, we expected a main effect of disease severity, such that MCID estimates would increase with greater disease severity, replicating findings of Andrews et al. [9]. Finally, it was expected that the influence of agreement would decrease with increasing severity of impairment, possibly due to anosognosia of the patient.

## Methods

### Sample

All necessary IRB approvals were obtained prior to the start of the study. All available data from the Uniform Data Set (UDS) was requested through the National Alzheimer’s Coordinating Center (NACC) on 8/10/2021. The UDS represents a standardized database with uniform collection of data across the Alzheimer’s Disease Research Centers, including cognitive assessments. The dataset included 43,999 participants from 39 Alzheimer’s Disease Research Centers. Diagnoses were made by a clinician or a consensus team, based on available interview, cognitive, behavioral, biomarker, imaging, and genetic data. Across the UDS sites, similar protocols were used for the interview and obtaining cognitive data; however, the protocols for collection of biomarker, imaging, and genetic data may have varied by the site. In order to determine a diagnosis using all available information, the following steps were done by the clinician or consensus team: (1) determination of whether the subject had healthy cognition; (2) if cognition was determined to be impaired, a diagnosis of dementia or mild cognitive impairment was made based on NACC consensus clinical criteria; (3) using clinical consensus criteria, a primary etiologic diagnosis for mild cognitive impairment or dementia was made.

**Fig. 1:**
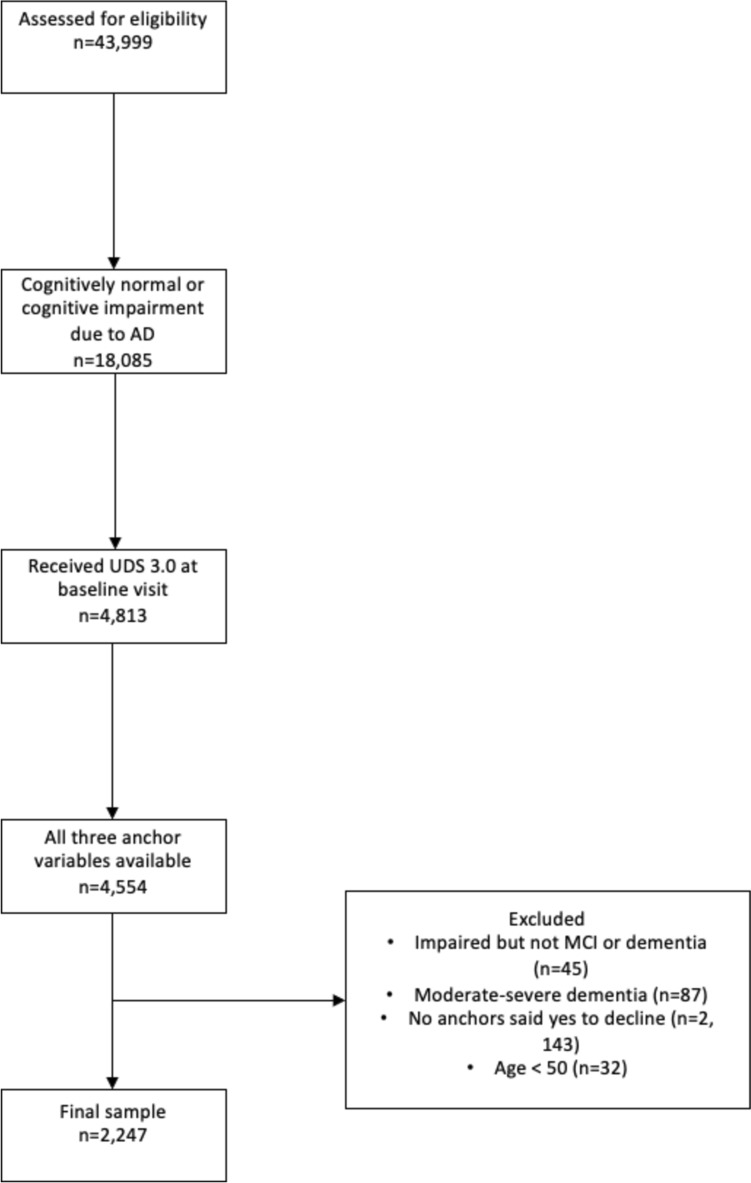
Strobe diagram of the selection process for the study

See Fig. 1 for an overview of our sample selection process.

Because of our interest in creating MCID estimates for AD, data was first reduced to individuals who were either cognitively normal or diagnosed with cognitive impairment due to AD.

Next, the sample was reduced to individuals who received the UDS 3.0 at their initial visit and also had at least one follow-up visit. Then we restricted the sample to only participants who had all three anchor variables available (participant anchor, co-participant anchor, and clinician anchor). In this sample, classification of cognitive status (cognitively normal vs. mild cognitive impairment vs. dementia) was based on UDS consensus decision during the participants’ initial visit. Individuals with dementia were further classified into severity categories based on the global Clinical Dementia Rating (1 = mild dementia, 2 = moderate dementia, 3 = severe dementia) [11]. Individuals with moderate and severe dementia were excluded due to small sample sizes, a high likelihood of difficulties participating meaningfully in cognitive evaluation [25–27], and the low prevalence of disagreement in terms of observed decline across the three anchors (n = 87). Since we were interested in calculating anchor-based MCID estimates, analyses were carried out in a sample of individuals who had at least one anchor answer affirmatively that they observed decline in the participant. Finally, individuals who were less than 50-years-old were excluded from the analyses (n = 2247), following the typical age cut-off in clinical trials used due to the low prevalence of cognitive disorders younger populations [28]. See Table 1 for final sample characteristics.

**Table 1:** Sample characteristics in the primary sample and across disease severity

	Primary sample	Cognitively normal	MCI	Mild AD	Moderate-severe AD
N	2247	685	751	811	85
Age (M, SD, range)	72 ± 8 (50–103)	71 ± 8 (51–103)	73 ± 8 (50–96)	71 ± 9 (50–98)	73 ± 12 (50–99)
Gender (n, %; F/M)	997 (44.4%)/1250 (55.6%)	438 (63.9)/247 (36.1)	380 (50.6)/371 (49.4)	432 (53.3)/379 (46.7)	51 (60.0)/34 (40.0)
Education (M, SD, range)	16 ± 3 (0–25)	16 ± 3 (1–25)	16 ± 3 (0–25)	15 ± 3 (0–25)	14 ± 3 (3–20)
Race/Ethnicity (n, %; Asian/Black/White/Hispanic)	57 (2.5)/253 (11.3)/1831 (81.5)/169 (7.5)	26 (4.0)/114 (16.6)/498 (72.7)/45 (6.5)	17 (2.2)/85 (11.3)/622 (82.8)/64 (8.5)	14 (1.7)/54 (6.7)/711 (87.7)/60 (7.4)	2 (2.4)/10 (11.8)/70 (82.4)/8 (9.4)
CDR-SB visit 1 (M, SD, range)	2.17 ± 2.14 (0–10)	0.24 ± 0.46 (0–3)	1.54 ± 1.10 (0–6)	4.38 ± 1.74 (0–10)	11.3 ± 2.15 (8.5–18)
CDR-SB visit 2 (M, SD, range)	3.09 ± 3.26 (0–18)	0.31 ± 0.59 (0–5.5)	2.25 ± 1.68 (0–11)	6.21 ± 3.10 (0–18)	13.2 ± 2.78 (6–18)
FAQ visit 1 (M, SD, range)	0.57 ± 0.86 (0–3)	0.01 ± 0.09 (0–1)	0.24 ± 0.48 (0–3)	1.35 ± 0.92 (0–3)	2.59 ± 0.57 (0–3)
FAQ visit 2 (M, SD, range)	0.84 ± 1.08 (0–3)	0.03 ± 0.22 (0–3)	0.49 ± 0.73 (0–3)	1.86 ± 0.98 (0–3)	2.78 ± 0.49 (0–3)
MoCA visit 1 (M, SD, range)	21.1 ± 5.9 (1–30)	26.2 ± 2.8 (11–30)	21.8 ± 3.6 (6–30)	15.8 ± 5.2 (1–29)	8.2 ± 4.8 (0–20)
MoCA visit 2 (M, SD, range)	20.2 ± 6.9 (0–30)	26.1 ± 2.9 (11–30)	21.0 ± 4.1 (7–30)	13.8 ± 6.2 (0–30)	6.3 ± 4.1 (0–17)

Primary sample refers to the sample used for primary analyses. FAQ data was missing for 28 participants. MoCA data was missing for 366 participants

### Measures for MCID estimation

CDR-SB and FAQ were selected based on prior work that provided MCID estimates for these scales [9]. It is relevant to also obtain MCID estimates for the MoCA due to frequent inclusion of cognitive screeners in clinical trials for the purposes of screening (e.g., [29]) and as an end point outcome (e.g., [30]).

#### MoCA

The Montreal cognitive assessment (MoCA) [15] is a brief screening measure for global cognition and includes multiple items sensitive to cognitive impairment, with scores ranging from 0 to 30. Assessed domains include memory, language, visuospatial abilities, executive functioning, attention, and orientation. Higher scores represent better performance.

#### CDR-SB

The clinical dementia rating scale-sum of boxes (CDR-SB, range: 0–18) [11] is a measure of clinical impairment related to cognitive aging and dementia. It is based on six cognitive and functional domains (memory, orientation, judgment, and problem solving, community affairs, home, and hobbies). Scores on these domains are summed to create the sum of boxes score. Higher scores represent greater degree of impairment.

#### FAQ

The functional activity questionnaire (FAQ) [12] is a measure of performance in instrumental activities of daily living. The FAQ is completed by a study partner and consists of 10 items asking about instrumental activities of daily living (e.g., traveling outside the neighborhood, organizing taxes and other records). Completion of each task is rated from 0 (no difficulty) to 3 (cannot do). Total FAQ score was calculated as a median score based on the individual’s available item responses to account for missing item responses. Higher scores represent greater degree of functional impairment in instrumental activities of daily living.

### Anchor variables

Anchor variables included responses to questions about cognitive decline of the participant, as rated by the participant, study partner, or clinician. The participant anchor variable consisted of a yes/no response to the question: “Does the subject report a decline in memory (relative to previously attained abilities)?” The study partner anchor variable consisted of a yes/no response to the question: “Does the co-participant report a decline in subject’s memory (relative to previously attained abilities)?” Finally, a clinician anchor variable was based on the yes/no response to the item: “Indicate whether the subject currently is meaningfully impaired, relative to previously attained abilities, in memory.” At participants’ subsequent NACC visit, “previously attained abilities” are defined relative to the previous visit.

### Analyses

#### Fleiss’ kappa

All analyses were conducted using R statistical software [31]. First, Fleiss’ kappa was used to measure agreement among the three anchors. Fleiss’ kappa was calculated for the overall agreement across anchors, as well as within each severity group. Fleiss’ kappa value of 0 indicates agreement as expected by chance. Scores from 0 to 0.20 indicate slight agreement, 0.21–0.40 indicate fair agreement, 0.41–0.60 indicate moderate agreement, 0.61–0.80 indicate substantial agreement, and 0.81–1.0 indicates almost perfect agreement [32]. Values less than 0 indicate disagreement among anchors with − 1.0 indicating perfect disagreement.

Next, anchor agreement was transformed into a dichotomous variable for purposes of the statistical analyses. Agreement about meaningful decline was calculated across anchors, the participant, study partner, and clinician. If all three anchors reported decline, the agreement variable was coded as ‘yes’ (1). If at least one anchor said there was no decline, the agreement variable was coded as ‘no’ (0).

#### MCID calculation

MCID estimates were calculated as average change from time 1 to time 2. Change in score for the CDR-SB, MoCA, and FAQ were calculated for each individual (second visit score minus first visit score).

#### ANOVA models

ANOVAs were conducted for each outcome variable, which included change scores on MoCA, CDR-SB, and FAQ. The main model was a 2 (between subjects) × 3 (between subjects) factorial ANOVA, including two independent variables: anchor agreement (yes/no) × cognitive status (cognitively normal vs. MCI vs. mild dementia). We examined all main effects and interactions. The agreement by cognitive status interaction provided information on how disease severity moderated the impact of anchor agreement on MCID estimates.

#### Power calculation

An a priori sensitivity analysis was performed to estimate the effect size that could be detected. Given sample size of 2247, the study was sufficiently powered (e.g., > 80%) to detect an effect size for partial η^2^ greater than or equal to 0.004.

## Results

### Anchor analysis—descriptive

**Fig. 2:**
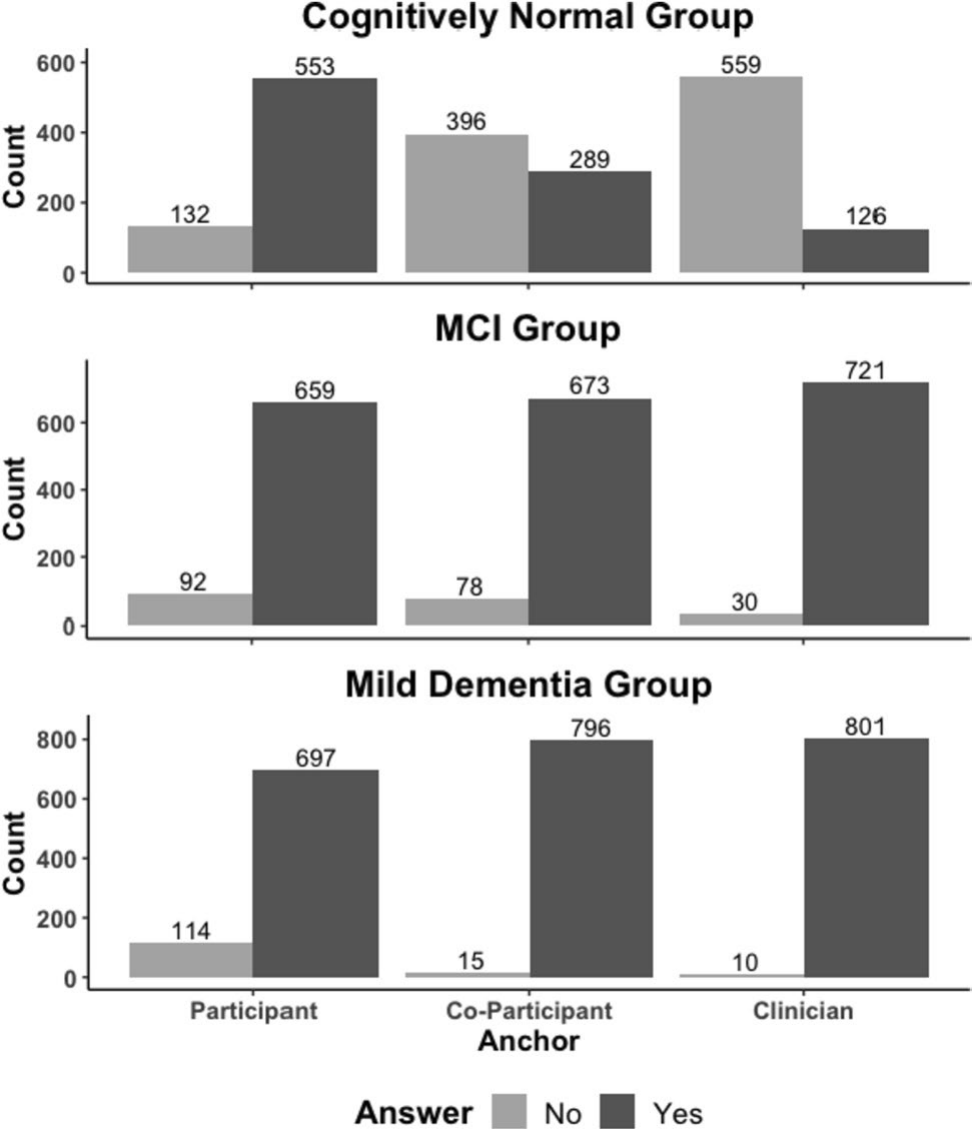
Number of individuals who responded No or Yes to the anchor questions across the severity groups

Anchor responses are visualized in Fig. 2. Agreement among the three anchors across severity groups was calculated using kappa. Overall, there was slight agreement among the anchors across the severity groups (kappa = 0.194, p < 0.001). Anchors showed significant disagreement in the cognitively normal group (kappa = − 0.21, p < 0.001) while anchors in the MCI group showed slight agreement (kappa = 0.144, p < 0.001). Finally, there was lack of agreement above chance in the mild dementia group (kappa = 0.0004, p = 0.982).

### Impact of anchor agreement and severity status on MCID estimation for the MoCA

**Fig. 3:**
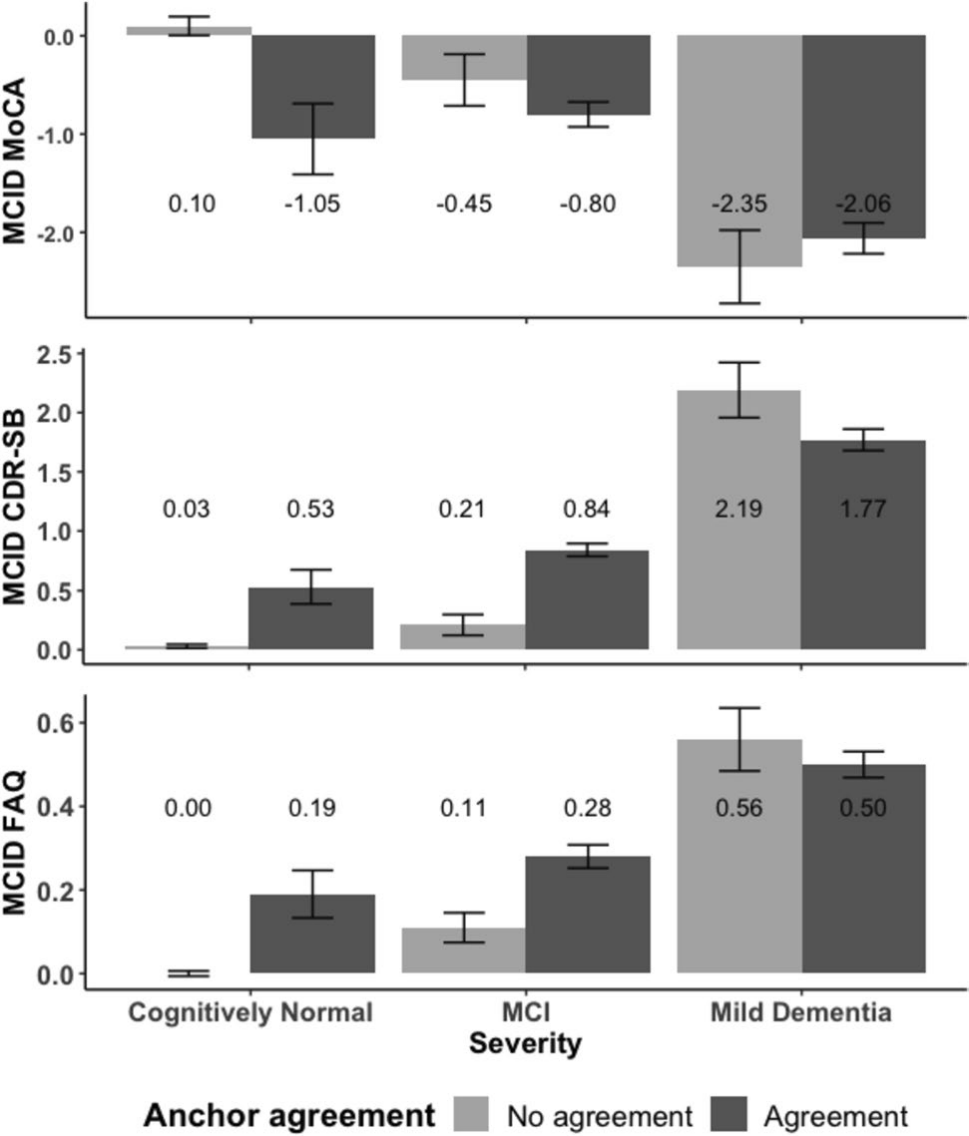
MCID estimates for MoCA, CDR-SB, and FAQ by anchor agreement and disease severity. Error bars represent standard error. Numbers represent MCID estimates. *MCID* Minimal clinically important difference, *MoCA* Montreal Cognitive Assessment, *CDR-SB* Clinical Dementia Rating-Sum of Boxes, *FAQ* Functional Assessment Questionnaire

To test study hypotheses, a dichotomous variable was used for anchor agreement in ANOVA models. Agreement significantly impacted MCID estimates (F(1,1875) = 60.761, p < 0.001; partial η^2^ = 0.03), such that the agreement group had higher MoCA change score (mean = − 1.42, SD = 3.28) than the no agreement group (mean = − 0.33, SD = 2.80; Tukey HSD p < 0.001). MCID scores were significantly different across the severity groups (F(2, 1875) = 50.131, p < 0.001; partial η^2^ = 0.05), such that decrease in MoCA was greatest for the mild dementia group (mean = − 2.11, SD = 3.66), then the MCI group (mean = − 0.73, SD = 2.87), and lowest for the cognitively normal group (mean = − 0.01, SD = 2.32; Tukey HSD: all p’s < 0.001). Finally, the influence of anchor agreement on MCID estimation for the MoCA was moderated by cognitive severity status (F(2, 1875) = 3.908, p = 0.020; partial η^2^ = 0.004). Post-hoc tests revealed that there was a significant difference between agreement groups in the cognitively normal group (p = 0.003; Cohen’s d = 0.50), but no difference in the MCI group (p = 0.203) and the mild dementia group (p = 0.451), see Fig. 3. The supplementary figures include descriptive MCID estimates for each anchor by endorsement vs. denial of decline across disease severities. Examination of Supplementary Fig. 1 shows that differences in MCID estimation in the cognitively normal group may have been driven by clinician raters.

### Anchor agreement and severity status on MCID estimation for the CDR-SB

Agreement significantly impacted MCID estimates (F(1, 2241) = 168.80, p < 0.001; partial η^2^ = 0.07), such that the agreement group had greater CDR-SB change score (mean = 1.29, SD = 1.98) than the no agreement group (mean = 0.37, SD = 1.38; Tukey HSD: p < 0.001). MCID estimates were significantly different across the severity groups (F(2, 2241) = 138.27, p < 0.001; partial η^2^ = 0.11), such that increase in CDR-SB was highest for the mild dementia group (mean = 1.84, SD = 2.42), then the MCI group (mean = 0.71, SD = 1.30), and lowest for the cognitively normal group (mean = 0.07, SD = 0.55; Tukey HSD; all p’s < 0.001). Finally, influence of agreement on MCID estimation for the CDR-SB was moderated by severity status (F(2, 2241) = 13.05, p < 0.001; partial η^2^ = 0.01). Post-hoc tests revealed that there was a significant difference between agreement groups in the cognitively normal (p < 0.001; Cohen’s d = 0.96) and MCI groups (p < 0.001; Cohen’s d = 0.49), but agreement did not impact MCID estimates for the mild dementia group (p = 0.065), see Fig. 3. Examination of Supplementary Fig. 2 shows that differences in MCID estimation in the cognitively normal group may have been driven by study partners and clinician raters. Since CDR-SB relies on the views of clinician, an additional dichotomous anchor agreement variable only included participant and study partner. Post-hoc analysis found that the main effects and interaction remained significant.

### Impact of anchor agreement and severity status on MCID estimation for the FAQ

Agreement significantly impacted MCID estimates (F(1, 2213) = 110.155, p < 0.001; partial η^2^ = 0.05), such that the agreement group had higher FAQ change scores (mean = 0.39, SD = 0.75) than the no agreement group (mean = 0.10, SD = 0.44; Tukey HSD: p < 0.001). MCID estimates were significantly different across the severity groups (F(2, 2213) = 61.214, p < 0.001; partial η^2^ = 0.05), such that increase in the MCID estimate for the FAQ was greatest for the mild dementia group (mean = 0.51, SD = 0.82), then the MCI group (mean = 0.24, SD = 0.64), and lowest for the cognitively normal group (mean = 0.02, SD = 0.21; Tukey HSD: all p’s < 0.001). Finally, the influence of anchor agreement on MCID estimation for the FAQ was moderated by cognitive severity status (F(2, 2213) = 4.939, p = 0.007; partial η^2^ = 0.004). Post-hoc tests revealed that there was a significant difference between agreement groups in the cognitively normal (p = 0.002; Cohen’s d = 0.90) and MCI groups (p < 0.001; Cohen’s d = 0.27), but no difference in the mild dementia group (p = 0.446), see Fig. 3. Examination of Supplementary Fig. 3 did not show clear differences in MCID estimation by individual raters. Since FAQ relies on the views of the study partner, an additional dichotomous anchor agreement variable only included participant and clinician. Post-hoc analysis found that the main effects remained significant. However, the influence of anchor agreement on MCID estimation for the FAQ was not moderated by cognitive severity status in this analysis (F(2, 2213) = 0.38, p = 0.686).

## Discussion

The aims of the current study were to (1) provide updated MCID estimates for measures of cognitive and functional outcomes in the most modern version of the Uniform Data Set (MoCA, CDR-SB, and FAQ), (2) to assess the influence of agreement among participant, informant, and clinician anchors on MCID estimation, and (3) to investigate whether disease severity moderates the impact of anchor agreement on MCID estimation. We hypothesized that there would be main effects of agreement on MCID estimates, such that when there is unanimous anchor agreement, there would be more change on outcome measures. We also hypothesized that there would be a main effect of disease severity on MCID, such that MCID estimates would increase with increasing levels of impairment. Lastly, we expected that the influence of anchor agreement on MCID estimation would decrease with increasing severity of cognitive and functional impairment.

### Anchor agreement about meaningful decline

Within our descriptive analyses, there was slight agreement among anchors about meaningful decline overall. This effect was largely driven by significant agreement in the MCI group. Perhaps unsurprisingly, there was significant disagreement for cognitively normal individuals. Many older adults express cognitive concerns, regardless of the presence of objective cognitive decline [33]; consequently, there are likely to be a high proportion of individuals who report cognitive declines that are not deemed as significant by outside observers. This finding suggests that anchor agreement is an important variable to consider in MCID estimation.

### The impact of anchor agreement on MCID estimation

In line with the findings of previous studies, we found that when anchors unanimously agreed about the decline, more change in cognition was observed over time (e.g., [23, 24]). The effect size for this relationship was small to medium. Given that different anchors have been shown to better capture cognitive changes at different stages of severity [19–22, 34–36], these findings support the conclusion that agreement about decline amongst anchors is associated with greater declines on outcome measures indexing cognitive and functional abilities. Hence, when only one anchor is used in MCID calculations, the MCID value likely represents an underestimate. To increase accuracy in MCID estimation, researchers likely need to use information from multiple anchors in anchor-based approaches to assessment of meaningful declines.

### The impact of cognitive status on MCID estimation

As expected, increasing levels of cognitive impairment were associated with increasing magnitude of the MCID for all three outcomes, replicating the results of Andrews et al. [9]. Depending on the disease severity, a 0.07–1.84 point increase on the CDR-SB, 0.02–0.51 point increase in median FAQ score, and 0.01–2.11 point decrease on the MoCA corresponded to a meaningful decline in functioning. Overall, the pace of cognitive decline increased with increasing severity of disease [37, 38]. Consequently, clinically meaningful declines in individuals with normal cognition are likely to be smaller than in individuals with mild cognitive impairment, who will in turn have smaller changes to quantify clinically meaningful differences than individuals with dementia. Results imply that evaluators should not take a “one size fits all” approach to evaluating MCIDs; rather, MCID estimates need to be stratified by disease severity to be appropriately interpreted. Of note, our MCID estimates tended to be lower than those put forth by Andrews et al. [9]. This inconsistency is likely explained by methodological differences. First, even though Andrews et al. also looked at two timepoints, they used multiple visits from each participant, while our study only examined two visits per participant to account for potential within-person dependency. Second, we utilized an updated version of the UDS.

### Interaction of anchor agreement and cognitive status on MCID estimation

For all three measures, there was also a significant interaction effect, such that with increasing disease severity, anchor agreement was less influential on MCID estimation. Post-hoc tests uncovered that the influence of agreement was significant for individuals with normal cognition for all three outcomes and for individuals with MCI for the FAQ and CDR-SB. However, agreement no longer had a significant impact on the MCID in the dementia group. One likely explanation of this interaction is that people with AD tend to lose insight about their own abilities as disease severity progresses [23, 24, 35]. Therefore, even if the person with dementia disagrees with the study partner and clinician, they may still exhibit declines in cognition and functioning that are evident on outcome measures. These results suggest that it might be more important to account for anchor agreement in the early disease stages for the anchor-based MCID estimation. In order to estimate whether aggregating across the anchors might bias the estimates, we also provided descriptive information on the MCID estimates broken down by the anchor across the disease severity categories, including moderate-severe dementia due to AD (see Supplementary Figs. 1–3). These results suggested that among cognitively normal individuals, the influence of agreement on MOCA and CDR-SB MCID estimates may have been driven by clinician raters (see Supplementary Figs. 1 and 2). These findings may imply an increase in predictive utility from expert judgments knowledgeable of subjective cognitive decline criteria [39].

## Limitations

There are several limitations to consider when evaluating these results. First, FAQ and CDR outcomes can be considered, to an extent, proxies for anchor responses, since they already incorporate views of the study partner and clinician, respectively. We attempted to control for this fact in our post-hoc analyses. Second, the anchor responses might not be completely independent of each other (e.g., the study partner may regularly hear the patient discuss their memory problems), since each anchor might be aware of the views of the other anchors, creating potential for bias. However, this bias is likely unavoidable in real world assessment scenarios involving older adults, study partners, and clinicians. A third limitation of this study is that the question assessing whether or not the anchor observed a decline in the participant’s memory was phrased slightly differently for the clinician compared to the participant and study partner. All questions referenced the participant’s memory and elicited a yes/no response from the anchor. However, the question for the participant and study partner asked about “a decline in memory (relative to previously attained abilities)”, while the question for the clinicians asked if the subject “is meaningfully impaired, relative to previously attained abilities, in memory”. This difference in phrasing may have influenced the way respondents answered the questions. Future studies might provide uniform phrasing for all anchors and address whether differences in phrasing can bias the MCID estimates in the anchor-based calculations. A fourth limitation is the calculation of MCID estimates for CDR-SB and MoCA in cognitively normal individuals. These measures may be less sensitive for detecting cognitive changes in this group. Future studies might examine other cognitive measures designed specifically for use among those without cognitive impairment (e.g., the Preclinical Alzheimer’s Cognitive Composite [40]), and the field would benefit from developing consensus on the most appropriate measures for use in the preclinical population. Nonetheless, it is important to note that we did detect statistically significant change on these measures within our cognitively normal sub-sample. Fifth, the MCID estimates were calculated based on UDS visits that were approximately one year apart. The length of time between assessments can influence amount of change in scores [41], such that the MCID estimates from this study may not apply when a different reassessment interval is used. Finally, while the UDS 3.0 sample is diversifying, it remains skewed toward non-Hispanic, white, and highly educated individuals, which may limit the generalizability of these findings. In the future, it would be beneficial to replicate these results with a more diverse population.

## Conclusion

In summary, the MCID for the MoCA, CDR-SB, and FAQ varied by anchor agreement and disease severity. Key findings were that (1) MCID estimates should be stratified by severity, and (2) MCID estimates should take into account ratings from multiple anchors, particularly when assessing change in cognitively normal individuals and those with MCI. This latter finding is of critical importance for emerging clinical trials, which overwhelmingly focus on the earlier stages of AD severity [42]. Furthermore, results indicate that regulators may wish to require meeting MCID thresholds that are specific to the chosen patient population and re-assessment window in order to demonstrate evidence of clinical significance.

### Supplementary Information

Below is the link to the electronic supplementary material.

Supplementary file1 (DOCX 361 KB)

## Data Availability

Data is freely available through the National Alzheimer’s Coordinating Center (NACC) https://naccdata.org/.
