# Supplementary material for: Clinical importance in Alzheimer’s disease: effects of anchor agreement and disease severity
Source: Aging Clin Exp Res. 2024 Jan 24;36(1):5. doi: 10.1007/s40520-023-02643-0 (PMC10808396; doi:10.1007/s40520-023-02643-0)
Supplement: Supplementary file 1 — Supplementary file1 (DOCX 361 KB) [file 40520_2023_2643_MOESM1_ESM.docx]

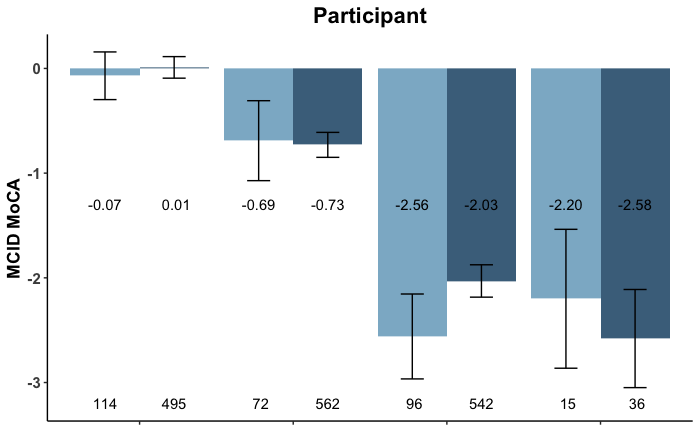


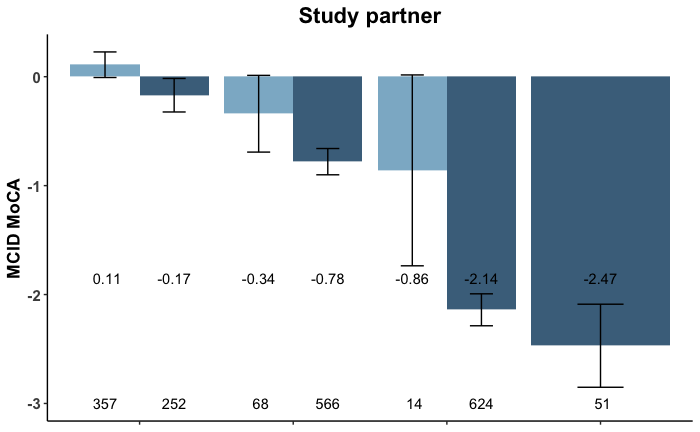


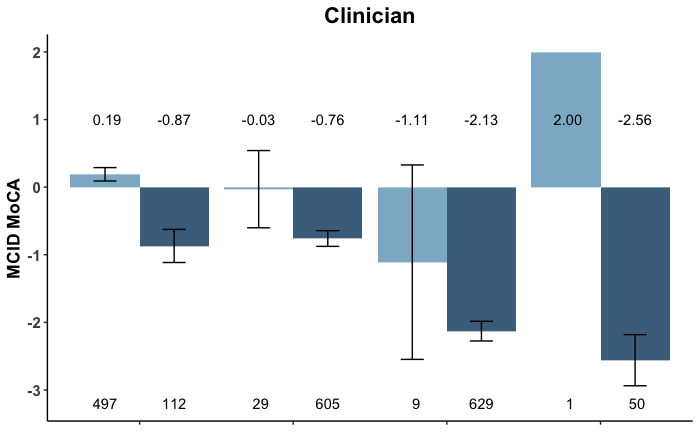


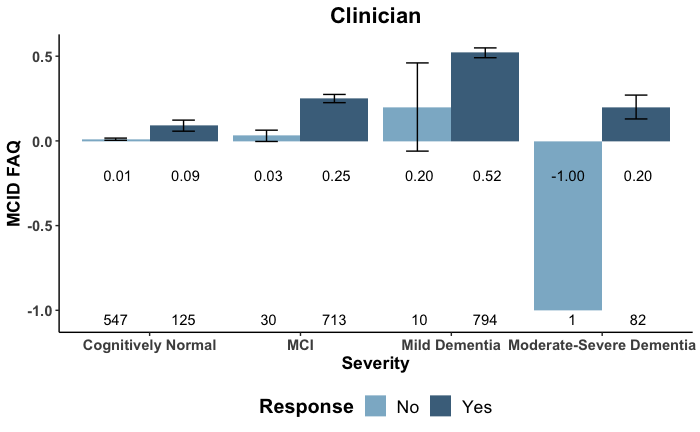


Supplementary Figure 1. Minimal clinically important difference (MCID) estimates for MoCA for each of the anchors divided by disease severity and whether they responded yes or no to the question about decline. The numbers at the x-axis represent sample size for each cell while numbers above represent MCID estimates. Error bars represent standard error.


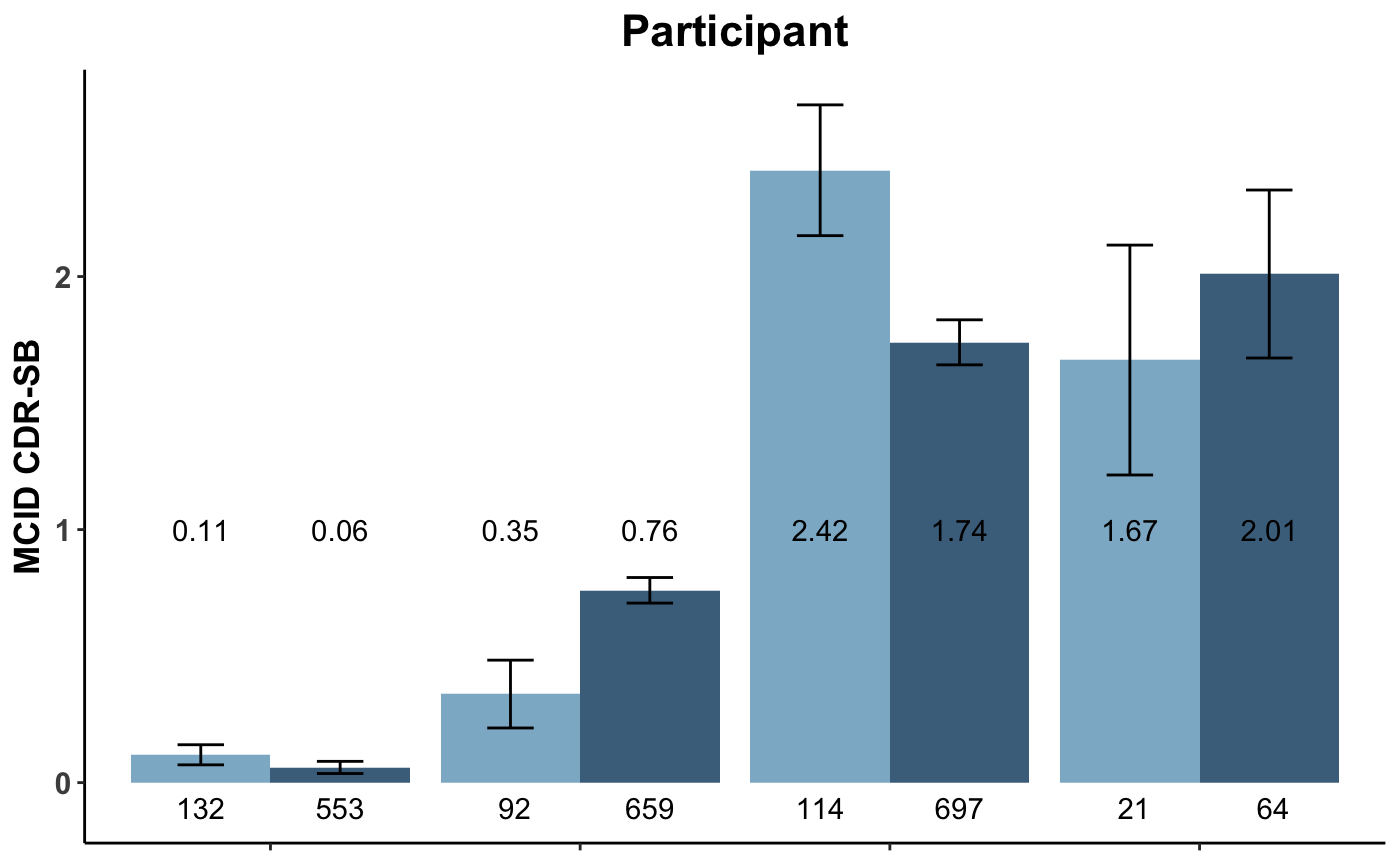


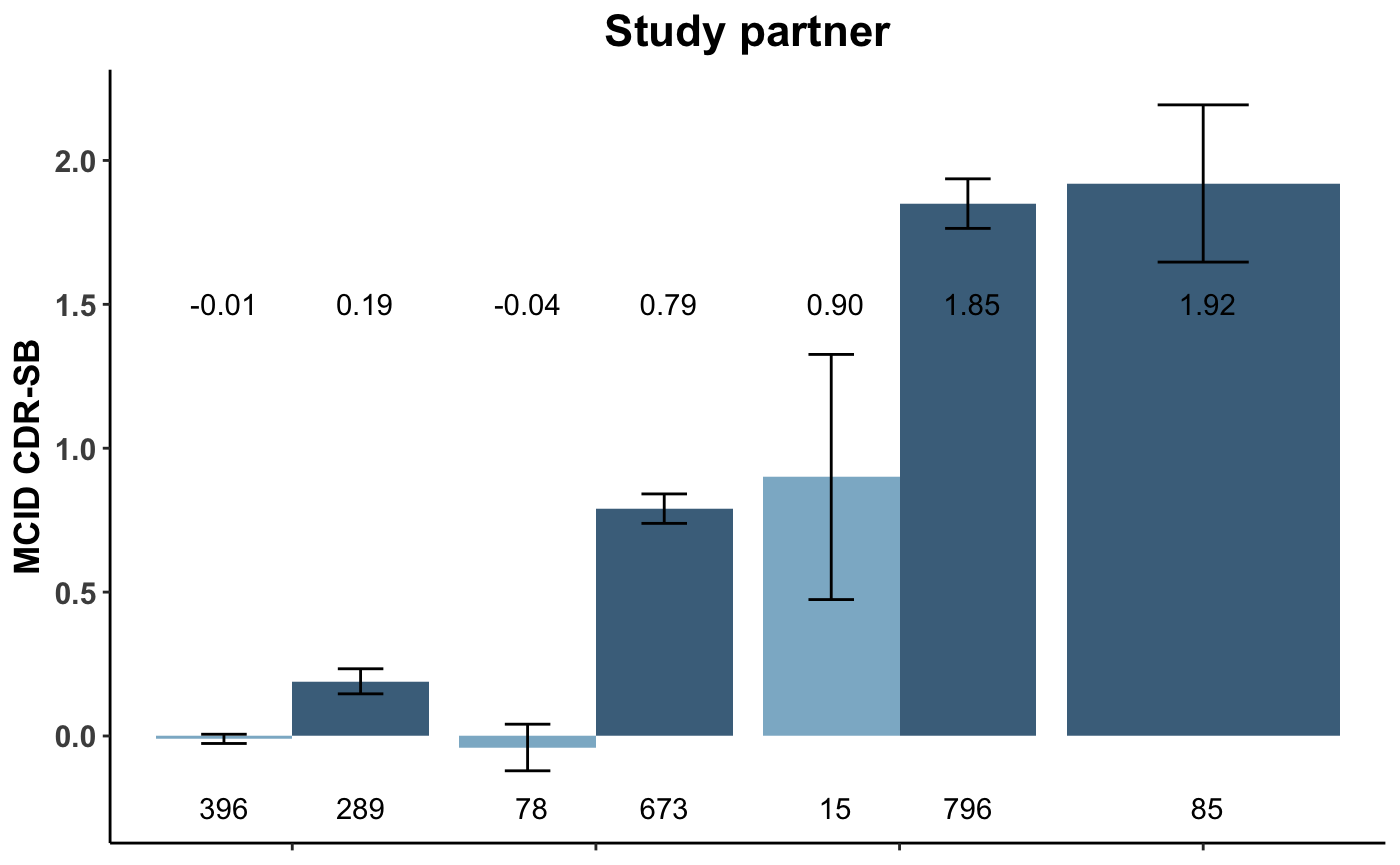


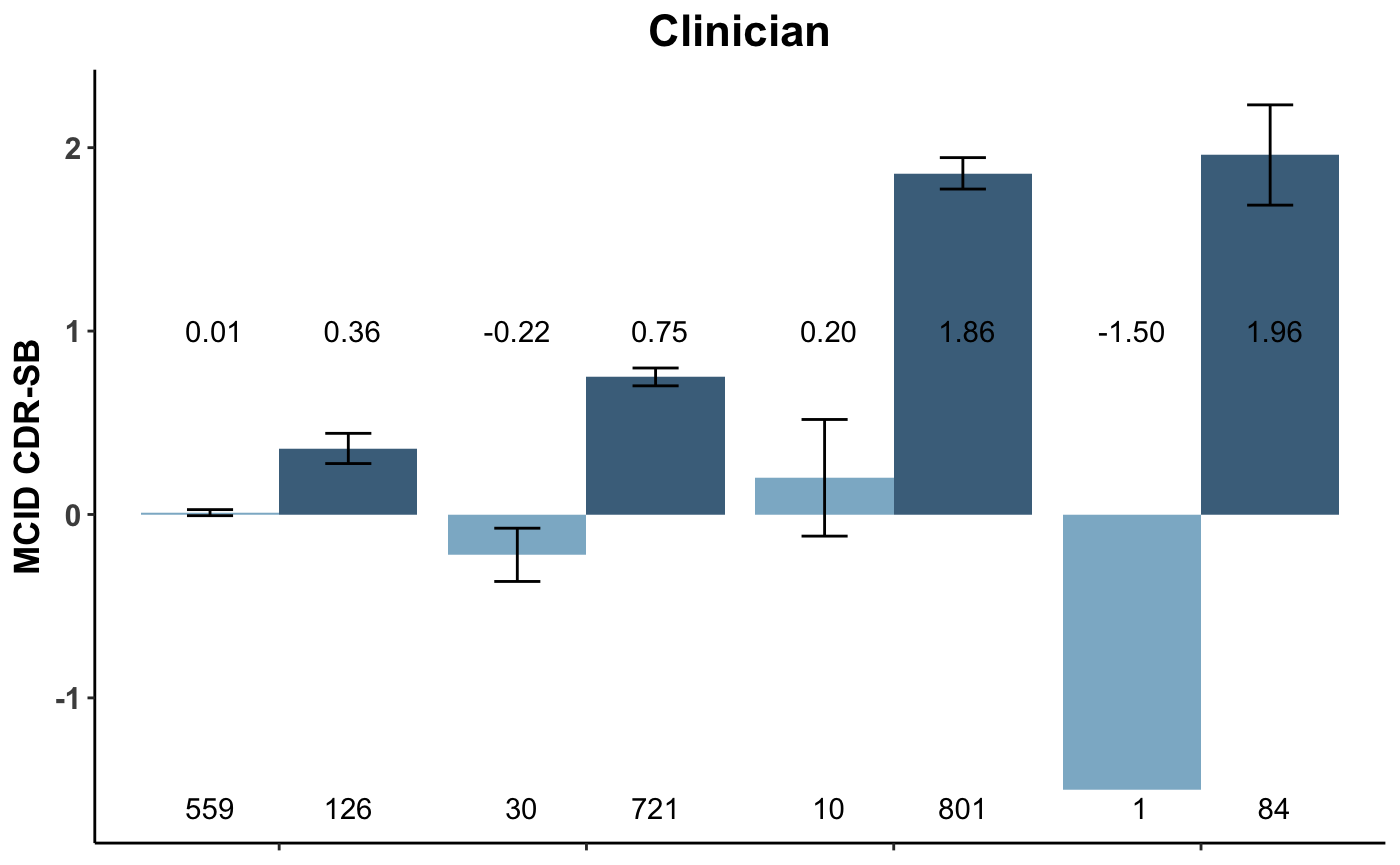


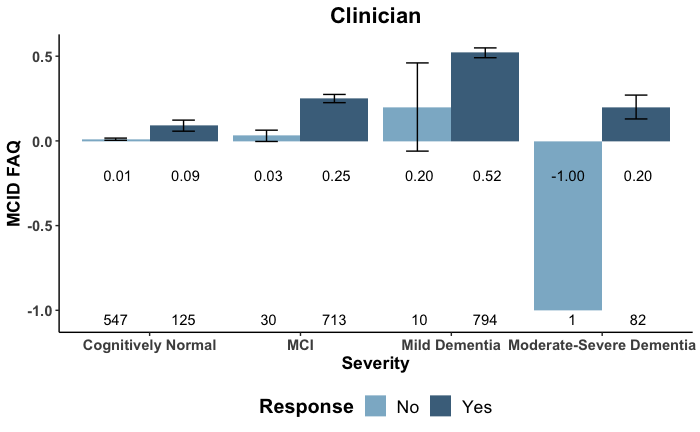


Supplementary Figure 2. Minimal clinically important difference (MCID) estimates for CDR-SB for each of the anchors divided by disease severity and whether they responded yes or no to the question about decline. The numbers at the x-axis represent sample size for each cell while numbers above represent MCID estimates. Error bars represent standard error.


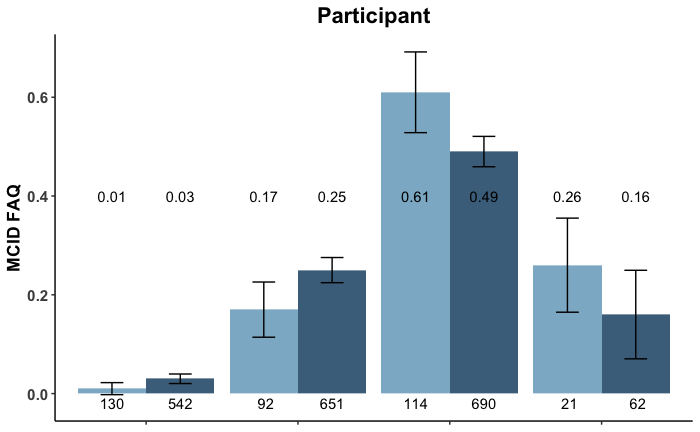


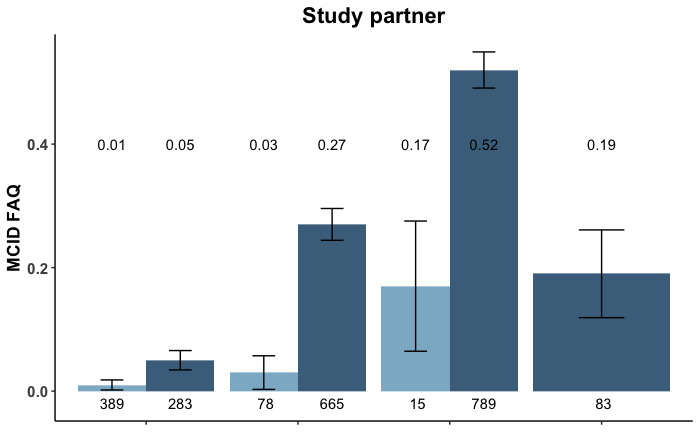


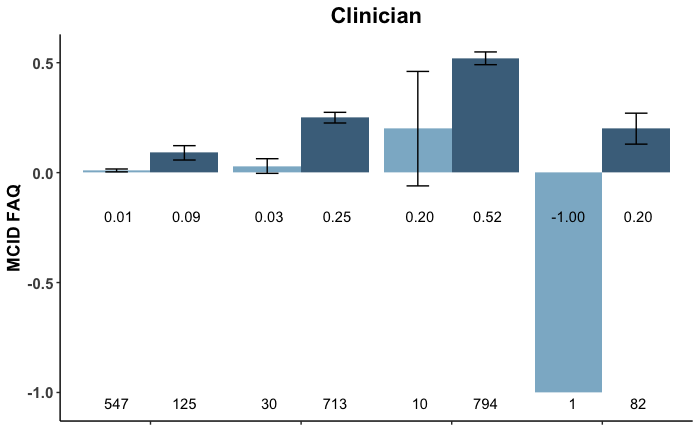


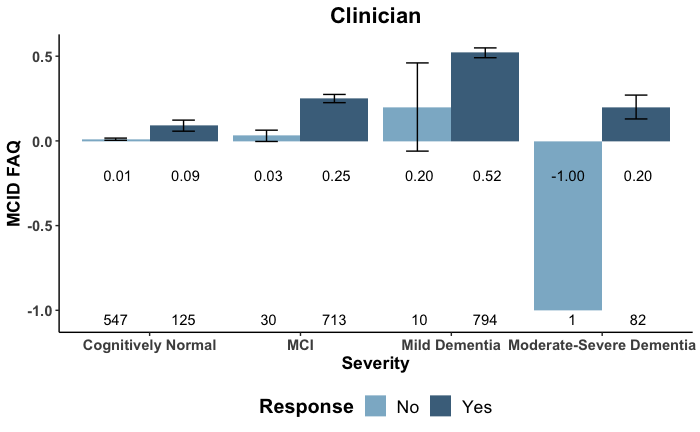


Supplementary Figure 3. Minimal clinically important difference (MCID) estimates for FAQ for each of the anchors divided by disease severity and whether they responded yes or no to the question about decline. The numbers at the x-axis represent sample size for each cell while numbers above represent MCID estimates. Error bars represent standard error.
